# Investigation of the female genital tract microbiome and its association with hydrosalpinx in women undergoing salpingectomy

**DOI:** 10.1007/s00404-025-07944-5

**Published:** 2025-03-14

**Authors:** Yael Yagel, Yair Motro, Stefan Green, Hadar Klapper-Goldstein, Ella Pardo, Jacob Moran-Gilad, Adi Y. Weintraub

**Affiliations:** 1 Department of Health Policy and Management, School of Public Health, Faculty of Health Sciences, Ben-Gurion University of the Negev, Beer Sheva, Israel; 2 Genomics and Microbiome Core Facility, Rush University Medical Center, Chicago, IL USA; 3 Department of Obstetrics and Gynecology, Soroka University Medical Center, Faculty of Health Sciences, Ben-Gurion University of the Negev, POB 151, Beer Sheva, Israel

**Keywords:** Microbiome, Fallopian Tubes, Hydrosalpinx, Salpingectomy

## Abstract

**Objective:**

To describe the microbiome of the vagina and fallopian tubes (FT) and its relation with hydrosalpinx.

**Methods:**

Case–control study was conducted in women who underwent salpingectomy for hydrosalpinx (case) or other indications (controls). Samples were obtained during surgery and subjected to 16S rRNA amplicon sequencing, and analyses of alpha diversity and beta diversity measures were compared between sites and groups. Differential abundance of bacteria associated with vaginal dysbiosis was compared between cases and controls.

**Results:**

Nine women with hydrosalpinx and 23 women without hydrosalpinx were included in the study. The mean age of studied women was 41 (range: 29–54) and most (89%) were premenopausal. After in silico decontamination, only 30% of control FT samples and 10% of case FT samples had evidence of bacterial presence. The vaginal microbiome of control patients showed greater abundance of lactobacilli, whereas the vaginal microbiome of case patients contained relatively more bacterial vaginosis-associated bacteria, such as *Prevotella* and *Atopobium*. A significant difference was found in alpha and beta diversity between vaginal and FT microbiomes in control patients as FT samples were more diverse. We found that women with hydrosalpinx had a more “dysbiotic” vaginal microbiome and in women without hydrosalpinx, microbial composition within the vagina and FT differed, possibly representing two distinct ecological environments.

**Conclusion:**

Women undergoing salpingectomy for various reasons harbored bacteria within their FT, while women with hydrosalpinx generally did not. This suggests that even though infection may be an underlying cause of hydrosalpinx, bacteria may not be present by the time patients require surgery.

**Supplementary Information:**

The online version contains supplementary material available at 10.1007/s00404-025-07944-5.

## What does this study adds to the clinical work

**Table:** 

Women with hydrosalpinx had less evidevnce of bacteria within their falopian tubes and a more dysbiotic vaginal microbiome compared with women without hydrosalpinx undergoing salpingectomy.	

## Introduction

The microbiota of the female genital tract and its role in health and disease has extensively been studied. The vaginal microbiome has been well described both in healthy reproductive-age women [1] and postmenopausal women [2]. Various community state types (CST) exist within the vaginal microbiome and while most states are dominated by lactobacilli, other diverse. Non-lactobacilli-based microbiome profiles have been described, sometimes associated with the presence of bacterial vaginosis (BV) [1, 3, 4].

Together with the well-characterized lower genital tract microbiome, the presence of bacteria in the upper reproductive tract including the uterine cavity, the placenta and fallopian tubes (FT), has been more frequently investigated in recent years [5]. Studies examining the presence of bacteria in the fallopian FT found a diverse microbial environment albeit in very low quantities [6–8]. A study examining the ovarian microbiome found significant differences between cancer patients and healthy controls [9]. As these sites are accessible only via a surgical procedure, there is an obvious obstacle in studying and establishing a healthy microbial fingerprint if one exists.

Hydrosalpinx denotes a blocked edematous FT that is filled with fluid. Tubal blockage and tubal infertility usually result from previous pelvic infection such as pelvic inflammatory disease (PID), but damage to the FT from previous surgery, endometriosis or adhesions can also result in hydrosalpinx [10, 11]. The association of hydrosalpinx with decreased pregnancy and implantation rates in in vitro fertilization (IVF) cycles has been widely reported and value for surgical treatments for tubal disease prior to IVF has been previously established [12].

Microbiome studies examining infertility found different endometrial and vaginal bacterial compositions associated with implantation and pregnancy rates [13], as well as different lactobacilli-dominated vaginal compositions associated with pregnancy rates in women with unexplained infertility [14]. BV has also been shown to be associated with increased risk for sexually transmitted infections [15], pelvic inflammatory disease [16] and preterm labor [17]. However, the association between the vaginal and FT microbiomes and their relation to hydrosalpinx has yet to be elucidated.

In this study, we aim to describe the vaginal microbiome of women undergoing salpingectomy for hydrosalpinx and compare it with those undergoing salpingectomy for various other indications. Furthermore, we analyze the microbiome of the resected FT and its association with hydrosalpinx.

## Methods

### Study design and population

The study was a prospective cohort study, performed at the Soroka University Medical Center, a 1100-bed tertiary hospital in southern Israel and was authorized by the institutional research ethics committee (0228-17-SOR). All women who planned to undergo elective salpingectomy were offered the opportunity to participate in the study. Women receiving immunosuppressive treatments were excluded from the study and women with overt active infections were excluded from the control group. After obtaining informed consent from willing participants, a vaginal swab was taken before local cleansing in the operating room following anesthesia. All women were treated with prophylactic antibiotics 30–60 min before the surgical incision. Samples were obtained by introducing nylon flocked swab (ESwab, Copan, Italy) to the vagina up to 5 cm from the introits via a speculum and rotational swabbing of the vaginal walls for 5–10 s. After laparoscopic resection of the FT, they were removed in a bag and placed on a sterile field. After changing gloves and opening a new scalpel, the surgeon opened the FT and sampled the FT cavity with the dedicated applicator.

Samples were immediately refrigerated and then frozen at − 80 °C within 24 h of sampling until further analysis. Metadata including: age, ethnicity, menopausal status and prior antibiotic treatments were extracted from patients’ medical records.

### DNA extraction, PCR amplification and sequencing

All samples were subjected to genomic DNA extraction, followed by 16S ribosomal RNA-based PCR amplification bacterial DNA sequencing and analysis. Genomic DNA was extracted from swabs using a LEV blood kit (Promega, Madison, WI) implemented on a Maxwell 16 instrument, following manufacturer’s protocol with several modifications. Modifications included a lysozyme incubation (10 ng/µL lysozyme; Thermo Fisher Scientific) for 30 min at 37 °C followed by bead-beating (40 s at 6 m/s) using a FastPrep-24 System (MP Biomedicals). Homogenized samples were transferred to the Maxwell cartridges for final purification of DNA.

Genomic DNA was prepared for sequencing using a two-stage amplicon sequencing workflow as described previously [18]. Initially, genomic DNA was PCR-amplified using primers targeting the V4 region of microbial 16S ribosomal RNA (rRNA) genes. The primers, 515F modified and 806R modified [19] contained 5′ linker sequences compatible with Access Array primers for Illumina sequencers (Fluidigm, South San Francisco, CA). PCRs were performed in a total volume of 10 µl using MyTaq^™^ HS 2X Mix (Bioline), primers at 500 nM concentration and approximately 1000 copies per reaction of a synthetic double-stranded DNA template (described below). Thermocycling conditions were as follows: 95 °C for 5′ (initial denaturation), followed by 28 cycles of 95 °C for 30 s, 55° for 45 s, and 72 °C for 30 s. One microliter of PCR product from each reaction was transferred to the second-stage PCR reaction. Each reaction was conducted in a final volume of 10 µl using MyTaq HS 2X mix and each well contained a unique primer pair of Access Array primers containing Illumina sequencing adapters, single index sample-specific barcode and linker sequences. Thermocycling conditions were as follows: 95 °C for 5′ (initial denaturation), followed by 8 cycles of 95 °C for 30 s, 60° for 30 s, and 72 °C for 30 s. Libraries were pooled and purified using 0.6X concentration of AMPure XP beads to remove short fragments below 300 bp.

### Bioinformatics analysis

Raw sequence data underwent quality control (QC), trimming, and removal of the synthetic DNA template using FastQC [20], trimmomatic [21] and bbmap (BBTools v37.28; [22]) respectively. The sequence data that passed QC were imported into the QIIME2 package (v2019.10) [23]) for analysis using the deblur pipeline [24] with the SILVA DB (v128 at 97%, [25]). Following filtering of chimeric sequences and taxonomic assignments, operational taxonomic units (OTUs) that were not assigned as bacteria were removed. Due to the nature of the samples (i.e., low biomass), additional filtering was conducted using the R package decontam with the function “isContaminant” (setting parameters: ‘method = ”prevalence”, neg = ”is.neg”, threshold = 0.1’) using the blank/negative control samples as negative control [26]. Finally, the samples were rarefied at a sampling depth of 4,000 using the diversity plugin (diversity core-metrics-phylogenetic). Differences in the relative abundance of microbial taxa between vaginal and FT samples were assessed using the R package microeco [27], with statistical significance measured with the Kruskal–Wallis statistical test (with Benjamini–Hochberg-adjusted *p* value < 0.05 considered significant). Alpha diversity (Shannon index) and beta diversity (Bray–Curtis dissimilarity index) metrics were measured using the QIIME2 diversity plugin (diversity alpha and diversity beta respectively). Comparisons of alpha diversity (Shannon index) between groups of interest (i.e., case vs. control, or vaginal vs. FT) were conducted using the QIIME2 diversity plugin (diversity alpha-group-significance), with the Kruskal–Wallis statistical test (with Benjamini–Hochberg-adjusted p values considered significant at < 0.05). Comparisons of beta diversity (Bray–Curtis dissimilarity) between groups of interest (as above) were conducted using the QIIME2 diversity plugin (diversity beta-group-significance) with the PERMANOVA statistical test (using 999 permutations, with Benjamini–Hochberg-adjusted *p* values considered significant at < 0.05). The linear discriminant analysis effect size (LEfSe) biomarker discovery tool [28] [used as part of the *R* package microeco] was used to identify differentially abundant OTUs where variation in abundance of taxa (with relative abundance greater than 0.01%) was considered significant where the LDA score was greater than 3 and/or the *p* value from the LEfSe test was < 0.05.

## Results

Thirty-two women were enrolled in the study, nine of whom underwent salpingectomy due to hydrosalpinx (cases) and 23 due to other indications (controls). One patient in the case group was later excluded from the analysis due to low material yield from all samples taken, as described below, leaving 8 patients in the case group. Table 1 describes the baseline characteristics of the study participants. The mean age of patients was 41, with women in the case group being slightly younger than controls (40 vs. 41.5). Of cases, 88% were premenopausal, vs. 91% of controls. Two ethnic groups were represented in this study, Jewish and Arabs, with no differences in their distribution between groups (25% Arabs in the cases and 26% in controls). Indications for surgery in the case group were either infection (4/8) or infertility (4/8). In the control group indications varied, the most common being sterilization (11/23) and leiomyomas (8/23). All of the women in the control group, and 75% of women in the case group underwent bilateral salpingectomy and samples were taken from both FT.

**Table 1:** Clinical and demographic characteristics of study participants

Patient #	Group	Age at operation	Ethnicity	Menopause	Contraceptives	Indication	Antibiotic-use
100	Case	47	Jewish	Post	None	Infection	Yes
101	Case	40	Jewish	Pre	Hormonal IUD	Infection	Yes
102	Case	29	Jewish	Pre	Unknown	Infertility	No
103	Case	Dropped from the analysis due to low yield sample					
104	Case	38	Bedouin	Pre	Oral	Infection	Yes
105	Case	36	Jewish	Pre	Unknown	Infertility	No
106	Case	44	Jewish	Pre	None	Infertility	Yes
107	Case	53	Bedouin	Peri	None	Infection	No
108	Case	33	Jewish	Pre	Oral	Infertility	No
200	Control	45	Jewish	Pre	IUD + oral	Leiomyoma	No
201	Control	42	Bedouin	Pre	None	Sterilization	No
202	Control	36	Jewish	Pre	IUD	BRCA	No
203	Control	41	Jewish	Pre	Hormonal IUD	BRCA	No
204	Control	40	Jewish	Pre	None	Sterilization	No
205	Control	46	Jewish	Pre	Hormonal IUD	Leiomyoma	No
206	Control	31	Jewish	Pre	None	Sterilization	No
207	Control	43	Jewish	Pre	None	Leiomyoma	No
208	Control	45	Bedouin	Peri	Hormonal IUD	Leiomyoma	no
209	Control	44	Jewish	Pre	Oral	Sterilization	No
210	Control	47	Jewish	Pre	None	Other	No
211	Control	45	Jewish	Peri	None	Leiomyoma	No
212	Control	43	Bedouin	Pre	IUD	Sterilization	No
213	Control	36	Bedouin	Pre	IUD + oral	Sterilization	No
214	Control	47	Bedouin	Post	None	Other	No
215	Control	37	Jewish	Pre	IUD	Sterilization	No
216	Control	39	Jewish	Post	IUD + oral	Sterilization	No
217	Control	44	Bedouin	Pre	None	Sterilization	No
218	Control	34	Jewish	Pre	Oral	Sterilization	No
219	Control	54	Jewish	Pre	None	Leiomyoma	No
220	Control	34	Jewish	Pre	IUD	Sterilization	No
221	Control	37	Jewish	Pre	None	Leiomyoma	No
222	Control	45	Jewish	Peri	IUD + oral	Leiomyoma	No

Ninety-four samples from 32 participants were available for sequencing, producing a total of 2,310,035 raw reads (Supplementary Table 1). After QC and in silico decontamination, 872,742 were available for analysis. After decontamination as described in the methods, 31/32 vaginal (V) samples (8/9 cases, and 23/23 controls) were available for further analysis, while only 10/62 FT samples belonging to 8/32 women (1/9 cases and 7/23 controls) produced sufficient genetic material for the final analysis (Supplementary Table 1). The total read count of the samples included in the final analysis was 787,362 with an average read count of 22,847 for V samples and an average read count of 7909 for FT samples (*p* < 0.001). The average read count for V samples of controls was 24,016, while the average read count for V sample of cases was 19,485 (*p* = 0.064). Since read counts are a surrogate marker for bacterial load, we further analyzed the average read count of case patients who received antibiotic treatment prior to sample collection and those who didn’t (24,005 vs. 14,964, *p* = 0.205). To account for the possible relation between vaginal read count and positive FT samples, we compared average vaginal read counts of patients with positive and negative FT samples and found no difference (25,091 vs. 23,569 respectively *p* = 0.079).

**Fig. 1:**
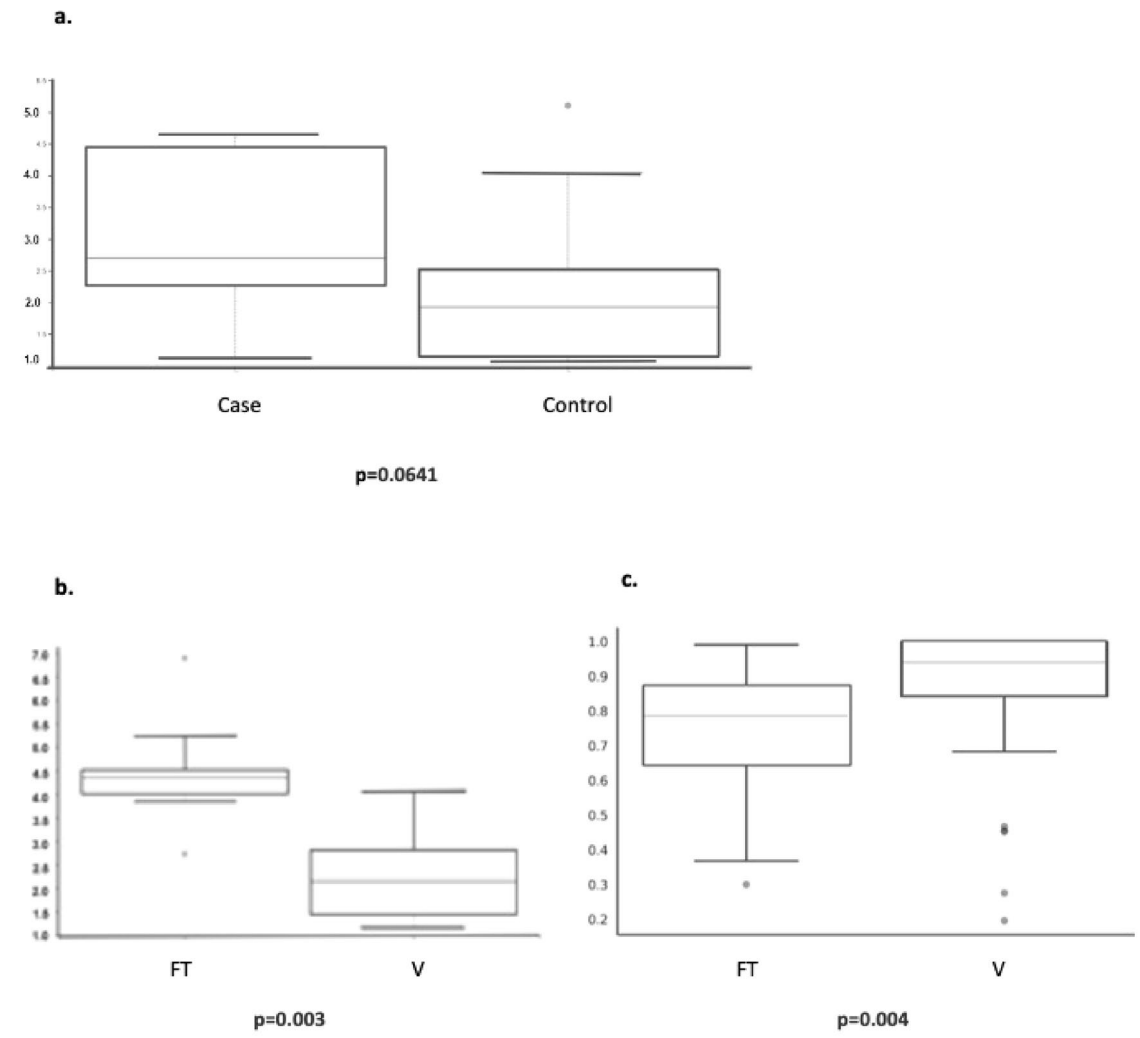
Diversity measures compared between sites and groups. **a** Alpha diversity plot for vaginal samples of cases vs. controls at the genus level (using Shannon index). **b** Alpha diversity plot comparing vaginal and FT samples of controls at the genus level (using Shannon index). **c** Beta diversity plot comparing vaginal and FT samples of controls at the genus level, (using Bray–Curtis dissimilarity index)

For analysis of OTUs and diversity measures, rarefaction was performed to a depth of 4000 sequences/sample as described in the methods, producing 145 total observed OTUs, ranging from 1 to 76 OTUs per sample with an average of 8.8 OTUs for V samples and 28.2 OTUs for FT samples (*p* < 0.001). Looking into the differences between vaginal samples of cases and controls, we examined the alpha diversity at the genus level as measured using the Shannon index. While the difference nearly reached statistical significance (*p* = 0.0641, Fig. 1a), the difference was further accentuated by comparing only vaginal case samples taken from patients who were operated due to infectio to control samples (none of the latter were operated due to infection) (*p* = 0.0243). These results remained statistically significant when analyzed at the phylum level. As most FT samples available after decontamination and rarefaction were from controls, we analyzed the differences in measures of diversity between matched vaginal samples of control patients to samples taken only from FT of the same control patients. Figure 1 illustrates the significant differences in both alpha (measured using the Shannon index, *p* = 0.003, Fig. 1b) and beta diversity (measured using Bray–Curtis dissimilarity index, *p* = 0.004, Fig. 1c) between both groups.

**Fig. 2:**
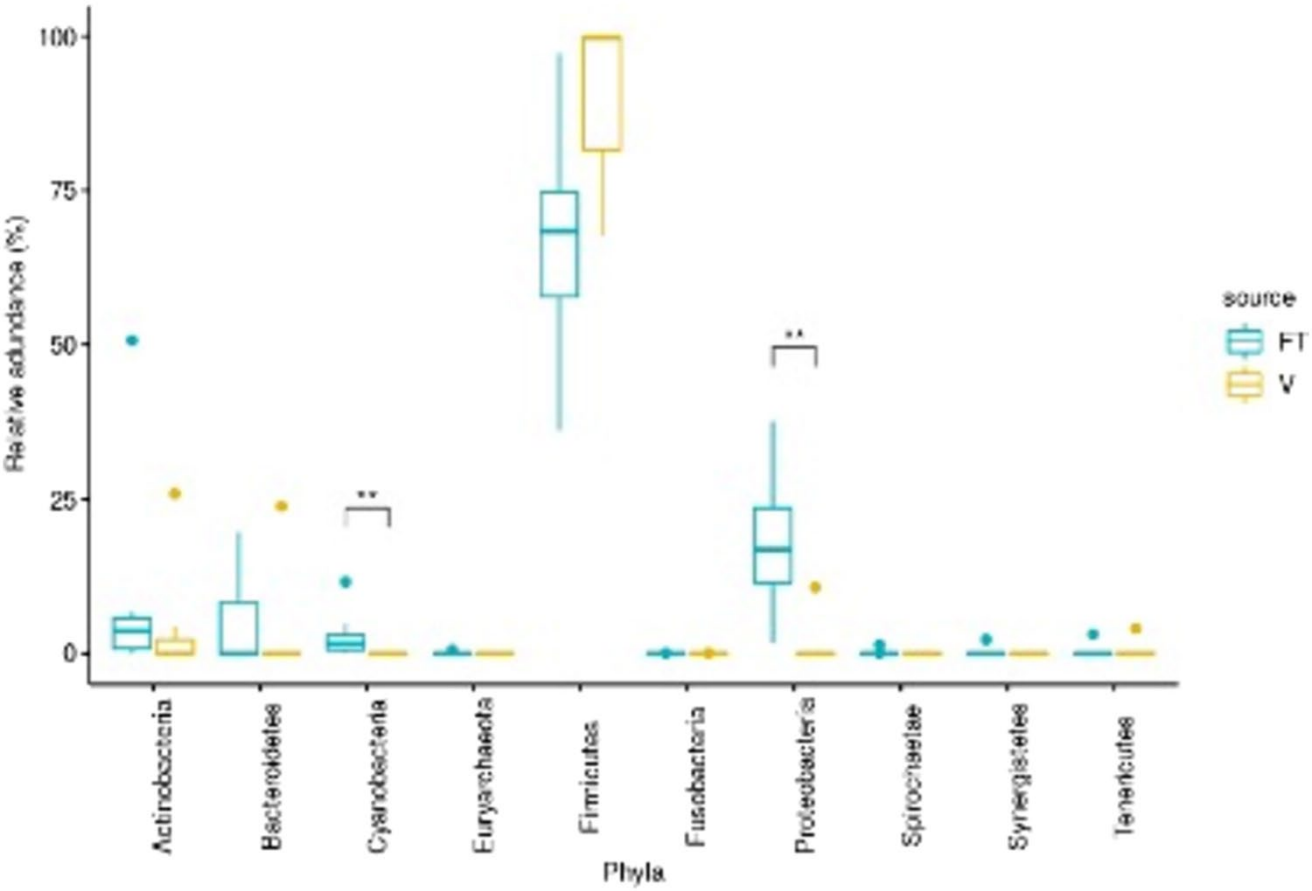
Relative abundances of different phyla in vaginal vs. FT samples

At the phylum level, both V and FT samples were composed mostly of Firmicutes, (83.1% in vaginal samples vs. 65.4% in FT samples *p* = 0.073) though the relative abundances of Proteobacteria and (18.3% vs. 2.16% respectively *p* = 0.011) and Cyanobacteria (0% in V, 2.73% in FT p = 0.011) in FT samples were significantly higher, as shown in Fig. 2.

**Fig. 3:**
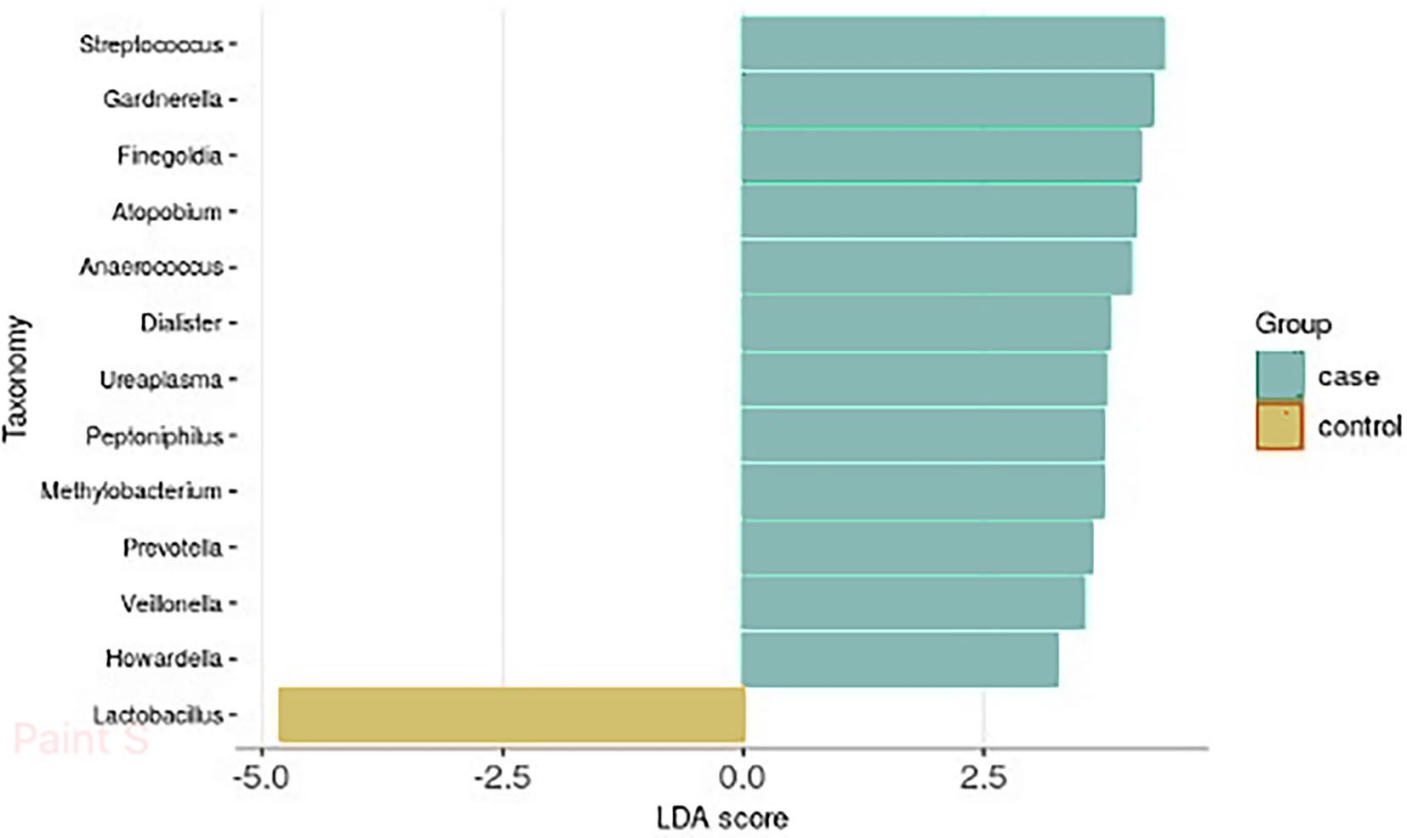
Differential abundances of bacterial genera in vaginal samples of cases and controls (using LEfSe)

Vaginal samples of both cases and controls contained mostly Firmicutes. While no significant difference was found in relative abundances of different genera, using linear discriminant analysis effect size (LEfSe) to account for differences in specific bacteria associated with vaginal dysbiosis, we found bacteria, such as *Prevotella*, *Gardnerella,* and *Atopobium*, to be more prevalent in case patients (*p* = 0.015, *p* = 0.013, *p* = 0.024 respectively), whereas Lactobacilli were more dominant in controls (*p* = 0.005), as shown in Fig. 3.

## Discussion

In our study, salpingectomy for various reasons was related to bacteria within the FT, while hydrosalpinx generally did not. In addition, women with hydrosalpinx show more “dysbiotic” vaginal microbiome, while in women without hydrosalpinx microbial composition within the vagina and FT differed both in composition and diversity of bacterial communities.

Several studies have shown the presence of bacterial DNA within the upper female reproductive tract [6–9]. However, a debate still exists regarding the presence of bacteria in sites considered normally sterile [29, 30]. A previous study comparing vaginal samples to FT samples found that the vagina contained four orders of magnitude more bacteria than the FT [7]. Another study found 20% of FT samples harbored no bacterial signal after PCR amplification and other samples contained varying and sometimes very low amounts of bacterial reads [8].

Our study demonstrated lower rates of positive FT samples in patients with hydrosalpinx. As this condition is sometimes associated with an infectious etiology, administration of extended antibiotic regimens instead of surgical resection has been attempted. Pathogenic bacterial presence has been hypothesized to be the cause of the deleterious effect of the stagnant fluid on fertility [32]. Our study results do not support this hypothesis since FT fluid of case patients didn’t contain bacteria, as opposed to controls, even in case patients who underwent surgery due to prior infection. When vaginal samples of patients with and without prior antibiotic treatment were compared, a non-significant difference in read counts was found. Nevertheless, when vaginal samples of patients with positive and negative FT samples were compared, there were no differences in average read counts, undermining the “quantitative” argument for this difference in bacterial presence. We believe that the vagina is the most important if not the sole source of bacterial colonization of the FT. Hence, we hypothesize that tubal occlusion resulting the hydrosalpinx is the cause of the paucity of bacterial presence within the FT, by blocking the anatomical pathway of bacterial migration. As this study included only nine patients with hydrosalpinx, it was underpowered to compare the results of women with or without prior antibiotic exposure. Such a comparison would have provided important data regarding the contribution of antibiotic treatment to FT bacterial colonization in the setting of tubal occlusion.

It is well accepted that different microbial profiles of the vagina are associated with disease states [4]. Looking into the differential abundance of the vaginal microbiota, the results marked differences in the bacterial composition of cases and controls. Figure 3 depicts the differences in the presence of certain bacteria associated with a dysbiotic vaginal environment between the two groups. Of these, *Gardnerella, Atopobium, Prevotella,* and *Ureaplasma* bacteria known to be dominant in bacterial vaginosis [33] and therefore infertility and adverse pregnancy outcomes were more prevalent in samples from case patients. Moreover, samples from case patients lacked the presence of lactobacilli, the most important genus associated with the normal vaginal microbiota, compared with controls. As stated above, case patients had higher rates of antibiotic exposure, which cannot be excluded as a contributing factor to the results. These findings, while not providing an answer to whether they are the cause or effect of the tubal occlusion, attest to the fact that women with hydrosalpinx are more likely to have a dysbiotic vaginal environment.

As samples that were taken from occluded FT in case patients did not contain enough bacterial DNA, after the decontamination process, we compared the microbiome of the vagina and fallopian FT only of matched control patients. As described in previously cited studies, these two environments were different in both alpha and beta diversity. The FT microbiome was found to be more diverse and while Firmicutes was the predominant phylum in both groups, FT samples were more abundant in Proteobacteria, similar to findings in previous studies. This highlights the fact that although bacteria in the FT may largely originate from the vagina, both sites may still represent different microbial niches.

In our study, we used stringent criteria to filter out possible contamination which led to the exclusion of 70% of FT samples in the control and 90% of FT samples in the case groups. These figures are in line with rates of culture-positive samples in other studies [7]. Exploring low-biomass samples is always subject to various contamination mechanisms [31] and the lower the amount of bacterial DNA present, the higher the relative weight any contamination bears on sample analysis. This was previously described by Miles et al. [6] who compared vaginal to FT samples, before and after exclusion of samples with containing low numbers of sequence reads. The exclusion of these samples enabled clustering of upper and lower genital tract samples more closely together. This may imply that some of the less abundant samples did not necessarily belong to actual anatomical microbiota. Furthermore, the genera described as more abundant in FT than in vaginal samples in other studies showed a clear trend toward environmental bacteria, such as *Acinetobacter, Pseudomonas,* and* Methylobacterium*. That is in contrast to culture-positive samples, which mostly contained bacteria which are typical residents of the vaginal microbiota. We believe that in the normal female reproductive tract, the major biological pathway of bacterial presence in the FT is the translocation of the vaginal microbiota through the upper genital pathway. This may account for the very low amounts of bacteria that were described. Thus, decontamination should be strongly considered when non-vaginal bacteria are found in FT samples.

Our study has several limitations. First, the small sample size limited us from performing subgroup analysis to various conditions that are known to affect microbial composition, such as menopausal state, age and former use of antibiotics. The latter is also a limitation in terms of the comparison of microbiota between cases and controls. Another limitation may be our decontamination protocols, which could be filtering out outputs that might have been of importance and the choice to set a high rarefaction cutoff in the setting of a low-biomass microbial environment causing many samples to fail the positivity criterion. While our preference was to optimize our analyzed data, it came with the cost of losing a substantial number of eligible samples. The study was performed at a single center, by the same surgical team which is an advantage when considering the consistency (internal validity) of the sampling technique in all patients, but its external validity and generalizability may be limited.

## Supplementary Information

Below is the link to the electronic supplementary material.

Supplementary file1 (DOCX 24 kb)
