# Supplementary material for: Investigation of the female genital tract microbiome and its association with hydrosalpinx in women undergoing salpingectomy
Source: Arch Gynecol Obstet. 2025 Mar 14;311(6):1649–56. doi: 10.1007/s00404-025-07944-5 (PMC12055863; doi:10.1007/s00404-025-07944-5)
Supplement: Supplementary file 1 — Supplementary file1 (DOCX 24 kb) [file 404_2025_7944_MOESM1_ESM.docx]

**Supplementary Table 1.** *Sample sequencing data*

|  | **sample-id** | **reads-raw** | **reads-deblur** | **reads-chimeric** | **reads-hit-reference** | **reads-missed-reference** |
| --- | --- | --- | --- | --- | --- | --- |
| **1** | CJH-203LF | 27 | 0 | 0 | 0 | 0 |
| **2** | CJH-205RF | 4134 | 1822 | 11 | 1674 | 116 |
| **3** | CJH-103LF | 1654 | 792 | 0 | 347 | 430 |
| **4** | CJH-222RF | 7356 | 3384 | 35 | 2833 | 437 |
| **5** | CJH-108LF | 6010 | 2617 | 32 | 1819 | 736 |
| **6** | CJH-219LF | 3588 | 1593 | 4 | 1477 | 90 |
| **7** | CJH-101RF | 7587 | 3360 | 17 | 2364 | 934 |
| **8** | CJH-201LF | 9574 | 4474 | 13 | 3187 | 1231 |
| **9** | CJH-215LF | 5431 | 2356 | 3 | 959 | 1347 |
| **10** | CJH-218RF | 3693 | 1669 | 0 | 601 | 1058 |
| **11** | CJH-211LF | 30662 | 14809 | 166 | 9786 | 4496 |
| **12** | CJH-216LF | 8339 | 3713 | 3 | 2986 | 690 |
| **13** | CJH-206RF | 11232 | 5194 | 17 | 4132 | 985 |
| **14** | CJH-208LF | 14392 | 6479 | 32 | 5581 | 814 |
| **15** | CJH-214RF | 11212 | 5100 | 18 | 2713 | 2303 |
| **16** | CJH-200RF | 10887 | 5082 | 21 | 3007 | 2005 |
| **17** | CJH-212RF | 10213 | 4898 | 26 | 3112 | 1727 |
| **18** | CJH-219RF | 10123 | 4727 | 14 | 2510 | 2163 |
| **20** | CJH-221LF | 9746 | 4563 | 21 | 2032 | 2477 |
| **21** | CJH-220LF | 10926 | 5226 | 0 | 2845 | 2358 |
| **22** | CJH-221RF | 17488 | 8490 | 28 | 6544 | 1868 |
| **23** | CJH-211RF | 16670 | 8008 | 37 | 4636 | 3244 |
| **24** | CJH-209RF | 10192 | 4845 | 0 | 2004 | 2788 |
| **26** | CJH-102RF | 15046 | 7023 | 27 | 3586 | 3331 |
| **27** | CJH-213RLF | 11726 | 5569 | 12 | 2359 | 3175 |
| **28** | CJH-217RF | 13946 | 6188 | 13 | 2287 | 3863 |
| **29** | CJH-201RF | 11828 | 5485 | 8 | 2068 | 3361 |
| **30** | CJH-200LF | 13416 | 6393 | 9 | 2806 | 3562 |
| **31** | CJH-103V | 12267 | 5635 | 10 | 1555 | 4041 |
| **32** | CJH-212LF | 22315 | 10548 | 17 | 7139 | 3306 |
| **33** | CJH-103RF | 6812 | 3184 | 4 | 709 | 2464 |
| **34** | CJH-105LF | 7595 | 3640 | 2 | 1141 | 2459 |
| **35** | CJH-205LF | 11534 | 5416 | 19 | 1203 | 4141 |
| **36** | CJH-100V | 23585 | 11148 | 51 | 5437 | 5598 |
| **37** | CJH-210RF | 25805 | 12113 | 33 | 8710 | 3299 |
| **39** | CJH-206LF | 15771 | 7250 | 26 | 2109 | 5045 |
| **40** | CJH-203V | 43699 | 22174 | 35 | 21974 | 22 |
| **41** | CJH-213RF | 20973 | 9888 | 19 | 3019 | 6805 |
| **42** | CJH-101V | 36430 | 17743 | 9 | 13571 | 4041 |
| **43** | CJH-210LF | 15375 | 7337 | 8 | 2461 | 4828 |
| **44** | CJH-215RF | 21804 | 10473 | 53 | 2874 | 7467 |
| **45** | CJH-102LF | 12443 | 5772 | 3 | 1013 | 4730 |
| **46** | CJH-202LF | 11203 | 5183 | 2 | 957 | 4211 |
| **47** | CJH-217LF | 19562 | 8764 | 4 | 1462 | 7235 |
| **48** | CJH-214LF | 19701 | 9433 | 16 | 4369 | 4993 |
| **49** | CJH-220RF | 13961 | 6639 | 0 | 1162 | 5453 |
| **50** | CJH-106V | 34784 | 17244 | 56 | 17076 | 4 |
| **51** | CJH-101LF | 13978 | 6708 | 15 | 1561 | 5106 |
| **52** | CJH-202RF | 14783 | 6934 | 2 | 1211 | 5691 |
| **53** | CJH-107LF | 12243 | 5707 | 2 | 1127 | 4536 |
| **54** | CJH-209LF | 21281 | 9977 | 22 | 2208 | 7684 |
| **55** | CJH-207RF | 11898 | 5712 | 20 | 1055 | 4633 |
| **56** | CJH-105RF | 17212 | 8299 | 17 | 1265 | 6963 |
| **58** | CJH-216RF | 18831 | 8816 | 18 | 1194 | 7543 |
| **59** | CJH-221V | 46331 | 24137 | 39 | 23992 | 10 |
| **60** | CJH-106LF | 15126 | 7091 | 11 | 1114 | 5924 |
| **61** | CJH-204LF | 14765 | 7058 | 3 | 925 | 6106 |
| **62** | CJH-207LF | 26411 | 12654 | 26 | 3377 | 9172 |
| **63** | CJH-218V | 32123 | 15946 | 19 | 15715 | 148 |
| **64** | CJH-108RF | 30869 | 14832 | 10 | 7683 | 7055 |
| **65** | CJH-222LF | 21863 | 10283 | 9 | 786 | 9461 |
| **66** | CJH-201V | 43419 | 22648 | 100 | 22395 | 99 |
| **67** | CJH-204RF | 18551 | 9039 | 22 | 559 | 8424 |
| **68** | CJH-218LF | 20143 | 9682 | 7 | 876 | 8765 |
| **69** | CJH-100RF | 22086 | 10947 | 5 | 1305 | 9615 |
| **70** | CJH-222V | 50830 | 26114 | 77 | 25694 | 234 |
| **71** | CJH-100LF | 19614 | 9562 | 14 | 418 | 9073 |
| **72** | CJH-104V | 49062 | 24349 | 40 | 23798 | 414 |
| **73** | CJH-203RF | 17768 | 8648 | 9 | 310 | 8292 |
| **74** | CJH-107RF | 19572 | 9411 | 3 | 371 | 9022 |
| **75** | CJH-104RF | 19358 | 9710 | 11 | 587 | 9091 |
| **76** | CJH-219V | 39930 | 20717 | 27 | 20621 | 32 |
| **77** | CJH-208RF | 47193 | 23209 | 49 | 21403 | 1682 |
| **78** | CJH-213V | 29252 | 15680 | 44 | 15591 | 28 |
| **79** | CJH-208V | 51467 | 26660 | 54 | 26481 | 57 |
| **80** | CJH-107V | 43515 | 23054 | 79 | 22913 | 25 |
| **81** | CJH-105V | 48506 | 24661 | 145 | 24413 | 96 |
| **82** | CJH-212V | 41945 | 22944 | 78 | 22780 | 35 |
| **83** | CJH-202V | 48123 | 25214 | 59 | 25095 | 47 |
| **84** | CJH-220V | 51442 | 26543 | 2 | 26328 | 198 |
| **85** | CJH-214V | 52388 | 26765 | 43 | 26589 | 121 |
| **86** | CJH-216V | 54162 | 24586 | 25 | 24493 | 58 |
| **87** | CJH-217V | 45218 | 23777 | 9 | 23651 | 109 |
| **88** | CJH-102V | 45832 | 24072 | 0 | 23816 | 249 |
| **89** | CJH-211V | 47955 | 26199 | 3 | 26168 | 9 |
| **90** | CJH-215V | 56644 | 30083 | 3 | 29653 | 390 |
| **91** | CJH-209V | 51653 | 28538 | 75 | 28438 | 19 |
| **92** | CJH-207V | 45663 | 24924 | 0 | 24868 | 45 |
| **93** | CJH-204V | 45698 | 24859 | 0 | 24691 | 160 |
| **94** | CJH-206V | 38253 | 21104 | 0 | 21061 | 38 |
| **95** | CJH-108V | 50364 | 24912 | 3 | 24881 | 24 |
| **96** | CJH-205V | 39663 | 22285 | 0 | 22265 | 11 |
| **97** | CJH-210V | 50062 | 28224 | 2 | 28213 | 7 |
| **98** | CJH-200V | 44273 | 25653 | 0 | 25636 | 13 |
